# Effects of fucoidan and synbiotics supplementation during bismuth quadruple therapy of *Helicobacter pylori* infection on gut microbial homeostasis: an open-label, randomized clinical trial

**DOI:** 10.3389/fnut.2024.1407736

**Published:** 2024-07-01

**Authors:** Huifen Wang, Wei Wei, Fang Liu, Miao Wang, Yanli Zhang, Shiyu Du

**Affiliations:** ^1^Department of Gastroenterology, China-Japan Friendship Hospital, Beijing, China; ^2^Department of Clinical Nutrition, Peking Union Medical College Hospital, Chinese Academy of Medical Science and Peking Union Medical College, Beijing, China

**Keywords:** *Helicobacter pylori* infection, quadruple therapy, fucoidan, synbiotics, gut microbiota

## Abstract

**Background:**

The eradication regimen for *Helicobacter pylori* (*H. pylori*) infection can induce gut dysbiosis. In this open-label, prospective, and randomized clinical trial, we aimed to assess the effects of fucoidan supplementation on the eradication rate and gut microbial homeostasis in the context of quadruple therapy, as well as to investigate the combined effects of fucoidan and synbiotics supplementations.

**Methods:**

Eighty patients with *H. pylori* infection were enrolled and randomly assigned to one of four treatment groups: the QT (a 2-week quadruple therapy alone), QF (quadruple therapy plus a 6-week fucoidan supplementation), QS (quadruple therapy plus a 6-week synbiotics supplementation), and QFS (quadruple therapy with a 6-week fucoidan and synbiotics supplementation), with 20 patients in each group. The QT regimen included rabeprazole, minocycline, amoxicillin, and bismuth potassium citrate. The synbiotics supplementation contained three strains of *Bifidobacterium*, three strains of *Lactobacillus*, along with three types of dietary fiber. All of the patients underwent ^13^C-urea breath test (^13^C-UBT) at baseline and at the end of the 6th week after the initiation of the interventions. Fresh fecal samples were collected at baseline and at the end of the 6th week for gut microbiota analysis via 16S rRNA gene sequencing.

**Results:**

The eradication rates among the four groups showed no significant difference. In the QT group, a significant reduction in α-diversity of gut microbiota diversity and a substantial shift in microbial composition were observed, particularly an increase in *Escherichia-Shigella* and a decrease in the abundance of genera from the Lachnospiraceae and Ruminococcaceae families. The Simpson index was significantly higher in the QF group than in the QT group. Neither the QS nor QFS groups exhibited significant changes in α-diversity or β-diversity. The QFS group was the only one that did not show a significant increase in the relative abundance of *Escherichia-Shigella*, and the relative abundance of *Klebsiella* significantly decreased in this group.

**Conclusion:**

The current study provided supporting evidence for the positive role of fucoidan and synbiotics supplementation in the gut microbiota. The combined use of fucoidan and synbioticss might be a promising adjuvant regimen to mitigate gut dysbiosis during *H. pylori* eradication therapy.

## 1 Introduction

*Helicobacter pylori* (*H. pylori*) infection is a global health problem, resulting in chronic inflammation and the increased risk of peptic ulcer disease, atrophic gastritis, gastric carcinoma, and mucosa-associated lymphoid tissue (MALT) lymphoma (1). The global pooled prevalence of *H. pylori* infection has been reported to be 48.5%, ranging from 70.1% in Africa to 24.4% in Oceania (2). A recent meta-analysis reported that the prevalence of *H. pylori* infection in mainland China was 40.0% during the period 2015–2019, highlighting China as one of the countries with a substantial burden of *H. pylori* infection, despite a declining trend from 2000 to 2019 (3). Besides, considering the high resistance rates of clarithromycin and metronidazole (4), bismuth quadruple therapy is recommended as the first-line treatment in China (5), including 2 antibiotics (with five different regimens), a standard dose of a proton pump inhibitor (PPI), and bismuth (6). Both antibiotics and PPIs are known to disrupt the gut bacterial equilibrium, and the bacterial shifts caused by eradication therapy may contribute to the disturbance of carbohydrate and lipid metabolism (7). Consequently, it is necessary to develop a therapeutic regimen for *H. pylori* infection that not only achieves a satisfactory eradication rate but also minimizes the impact on the gut microbiota.

Fucoidan is a complex sulfated polysaccharide extracted from various species of brown seaweed (8). There is a growing body of evidence suggesting that fucoidan possesses various physiological properties, including high antioxidant activity (9), anti-tumor effect (10), and anti-allergic property (11). Besides, fucoidan can exert anti-inflammatory effects by modulating neutrophil migration and the expression of genes related to COX-2 and the NF-κB signaling pathway (12). Therefore, fucoidan is regarded as a promising component in functional food or adjuvant therapy. Fucoidan has two primary structural backbones: Type I, such as fucoidan from *Laminaria japonica*, is composed of repeated α (1 → 3)-linked α-L-fucopyranose, with fucose-linked sulfate groups in C2- and C4- positions; Type II, such as fucoidan from *Ascophyllum nodosum*, is composed of alternate α (1 → 3)- and α (1 → 4)-linked α-L-fucopyranose, with fucose-linked sulfate groups in C2-, C3-, and C4-positions (13). The sulfate groups are important determinants for the bioactivities of fucoidan, depending on the sulfate content and position of the groups (14).

Research in a rat model of breast cancer has indicated that fucoidan intervention can enhance the diversity of the intestinal microbiota and change the microbiota composition, including a decrease in the abundance of *Collinsella*, *Coprococcus*, *Oscillospira*, and *Sporosarcina* as well as an increase in the abundance of *Parabacteroides* and *Blautia* (15). In an ulcerative colitis mice model, fucoidan was also found to significantly increase the abundance of Ruminococcaceae and *Clostridium-IV* (16). Moreover, fucoidan has been demonstrated to increase several beneficial bacterial taxa and decrease several harmful taxa in mice intervened with ciprofloxacin-metronidazole (17), indicating a potential capacity to mitigate dysbiosis caused by antibiotics, which is common during the eradication therapy of *H. pylori* infection. Nevertheless, data on the effects of fucoidan on the human gut microbiota are limited. In patients with chronic superficial gastritis, a combination of wheat peptides and fucoidan was reported to relieve symptoms, reduce gastric mucosal damage, and regulate gut microbiome, promoting the growth of *Bifidobacterium pseudocatenulatum*, *Eubacterium siraeum*, and *Bacteroides intestinali* (18), but the isolated effects of fucoidan were not evaluated.

Both *in vitro* and *in vivo* studies have shown that fucoidan can prohibit *H. pylori* adhesion to host cells, reduce cytotoxic effects in *H. pylori*, and decrease the infection rate of *H. pylori* in a dose-dependent manner (19). However, clinical data regarding the effects of fucoidan on *H. pylori* infection remains limited. Thus, we conducted a prospective, randomized, open-label trial in patients with *H. pylori* infection. The aim was to assess the effects of fucoidan supplementation, either alone or in combination with synbiotics supplementation, on the eradication rate and the maintenance of gut microbiota homeostasis in these patients.

## 2 Materials and methods

### 2.1 Subjects

This clinical trial was conducted at China-Japan Friendship Hospital from January 2021 to August 2021. *H. pylori*-infected patients aged above 18 years were enrolled. The infection of *H. pylori* was diagnosed for the first time in these patients based on a positive ^13^C-urea breath test (^13^C-UBT) and/or detection of *H. pylori* on gastric specimens collected during gastroscopy. All *H. pylori*-infected patients enrolled in this study were treatment-naïve. Patients would be excluded from this study if they fulfilled any of the following criteria: prior eradication therapy for *H. pylori* infection; peptic ulcer or other upper gastrointestinal lesions; upper gastrointestinal malignant tumor; acute or chronic lesions of major organs (e.g., cardiac, pulmonary, hepatic, or renal diseases); endocrine and metabolic disorders (e.g., diabetes mellitus, thyroid diseases); autoimmune diseases; use of antibiotics, antacids, bismuth, probiotics, prebiotics, or synbioticss within the previous 4 weeks; and pregnancy or lactation. This study was approved by the Ethics Committee of China-Japan Friendship Hospital (2020-64-K35) and was registered on the Chinese Clinical Trial Registry, number CTR2400080402. All participants provided their informed consent before participating in the study.

### 2.2 Study design

All patients underwent ^13^C-UBT at baseline, and then a computer-generated list of random numbers using SAS 9.0 (SPSS Institute, Cary, NC, United States) was used to randomly assign the patients to four treatment groups, and the corresponding intervention began immediately: (1) the QT group, receiving bismuth quadruple therapy, including rabeprazole 10 mg, minocycline 100 mg, amoxicillin 1,000 mg, and bismuth potassium citrate 220 mg, all administered twice daily for 2 weeks, namely the RMAB regimen; (2) the QF group, receiving RMAB therapy along with fucoidan 1 g once daily for 6 weeks, with fucoidan supplementation extending 4 weeks beyond the completion of quadruple therapy; (3) the QS group, receiving RMAB therapy along with a packet of synbiotics once daily for 6 weeks; and (4) the QFS group, receiving RMAB therapy along with 1 g fucoidan and a packet of synbiotics daily for 6 weeks. To assess the eradication of *H. pylori*, all of the patients underwent ^13^C-UBT 4 weeks after the completion of quadruple therapy. To evaluate possible side effects of interventions, new-onset symptoms during the 6 weeks were recorded.

The fucoidan used in this study was extracted from *Laminaria japonica* (Qingdao Brightmoon Seaweed Group Co., Ltd., Qingdao, China), with an average molecular weight of 276,038 Da and a purity of 90%. The monosaccharide fucose and the sulfuric acid group contents were 22.66% (w/w) and 29.65% (w/w), respectively. The prebiotics in the synbiotics used in this study were *Bifidobacterium* (accounted for 30%, including *B. animalis BB12*, *B. lactis Bi07*, and *B. bifidum Bb06*) and *Lactobacillus* (accounted for 70%, including *L. helveticus R52*, *L. rhamnosus R11*, and *L. acidophilus NCFM*), with a total colony forming unit (CFU) reaching 18 × 10^9^ in each packet; The prebiotics in the synbiotics packet included resistant dextrin 0.5 g, fructooligosaccharides 0.3 g, and inulin 1.0 g (Yu Jun Gong Wu^®^, lyophilized powder, Baoding Xiongnan Biotechnology Co., Ltd., Baoding, China).

### 2.3 16S rRNA gene sequencing

Fresh stool samples were collected with sterile plastic tubes from enrolled patients at baseline and at the end of 6th week in China-Japan Friendship Hospital. All of the samples were immediately transferred to the laboratory and stored at −80°C until analysis. Microbial DNA was extracted from the fecal samples using a QIAamp PowerFecal DNA Kit (QIAGEN, Germany). The amplification of the hypervariable V3-V4 region was conducted using primers 341F (5′-CCTACGGGNBGCASCAG-3′) and 805R (5′-GACTACNVGGGTATCTAATCC-3′). PCR reactions were performed in 25 μL mixture containing 5 μL of 5 × GC Buffer, 0.5 μL of KAPA dNTP Mix, 0.5 μL of KAPA HiFi HotStart DNA Polymerase, 0.5 μL of each primer (10 pM) and 50 ~ 100 ng of template DNA. After purifying the 16S V3 and V4 amplicon away from free primers and primer dimer species, PCR reactions were performed in 25 μL mixture containing 5 μL of 5 × GC Buffer, 0.75 μL of KAPA dNTP Mix, 0.5 μL of KAPA HiFi HotStart DNA Polymerase, 1.5 μL of each primer (10 pM) and 5 μL of purified product. The amplicons were subsequently purified by AMPure XP beads to construct the final library. Then sequencing was performed using the Illumina Hiseq platform (Illumina, CA, United States).

### 2.4 Analysis of gut microbiota composition

Fast Length Adjustment of SHort reads (FLASH) was used to merge paired-end reads from next-generation sequencing. The sequences were grouped into operational taxonomic units (OTUs) with a 97% threshold of similarity. Alpha diversity was analyzed with QIIME (V1.9.1), including the Shannon index and Simpson index to depict microbial diversity as well as the Chao1 index and observed species to depict microbial richness. Beta diversity was assessed based on weighted Unifrac distance matric, and principal coordinate analysis (PCoA) was conducted to visualize the distance between data. Analysis of similarities (Anosim) was used to evaluate the differences in microbial composition before and after interventions or between groups. The comparison of relative abundance bacterial taxa between groups was performed by Wilcoxon–Mann–Whitney test. The distinguishing features of the fecal microbiota were analyzed using linear discriminant analysis (LDA) effect size (LEfSe), and differences with log 10 LDA scores (absolute values) > 2.0 and *p* value < 0.05 were considered statistically significant. Meanwhile, metastats was used to identify the taxa with significantly different abundances between groups.

### 2.5 Statistical analysis

Statistical analyses were performed using SPSS version 22.0 (SPSS Inc., Chicago, IL, United States). Comparisons of continuous parameters were performed using independent samples *t*-test between two groups or one-way ANOVA among multiple groups, and comparisons of non-normal distribution data were performed using Wilcoxon–Mann–Whitney test or Kruskal-Wallis test. Categorical data were described as percentages and were compared using the chi-squared test or Fisher’s exact test. A two-sided *p* < 0.05 was considered statistically significant.

## 3 Results

### 3.1 Baseline demographics and eradication rate

A total of 80 patients were enrolled in this study and randomly assigned to receive either QT, QS, QF, or QFS therapy, with 20 patients in each group. The sex distribution and age of the patients in the four groups showed no significant difference. All of the participants fully completed the therapies and underwent ^13^C-UBT at week 0 and week 6. The QT, QS, QF, and QFS eradication rates were 95.0, 90.0, 100.0, and 95.0%, respectively, showing no significant difference. Detailed demographic characteristics and eradication rates are presented in Table 1. Several new-onset gastrointestinal symptoms were reported by patients, without significant differences among groups, indicating that the use of fucoidan or synbiotics neither improved nor exacerbated new-onset gastrointestinal symptoms during eradication therapy of *H. pylori* infection (Table 2).

**Table 1:** Demographic characteristics and eradication rates of *Helicobacter pylori* infection.

	QT group	QF group	QS group	QFS group	*p* value
Patients number	20	20	20	20	NA
Age in yr	39.30 ± 12.38	36.90 ± 8.77	39.25 ± 11.24	37.05 ± 9.48	0.943
Gender (Male: Female)	7:13	11:9	9:11	7:13	0.522
Eradication rate	95.0% (19/20)	100.0% (20/20)	90.0% (18/20)	95.0% (19/20)	0.899

The age data are presented as the mean ± SD; QT group, bismuth quadruple therapy for 2 weeks; QF group, bismuth quadruple therapy for 2 weeks combined with fucoidan for 6 weeks; QS group, bismuth quadruple therapy combined with synbiotics for 6 weeks; QFS group, bismuth quadruple therapy combined with both fucoidan and synbiotics for 6 weeks; NA, not applicable.

**Table 2:** Patients reporting new-onset gastrointestinal symptoms in different groups.

	QT group	QF group	QS group	QFS group
Patients number	20	20	20	20
Belching	1 (5%)	1 (5%)	1 (5%)	3 (15%)
Heartburn	2 (10%)	0	0	0
Nausea	0	1 (5%)	0	0
Barborygmus	0	2 (10%)	1 (5%)	2 (10%)
Abdominal distension	3 (15%)	2 (10%)	1 (5%)	0
Abdominal pain	1 (5%)	0	0	0
Increased defecation frequency	3 (15%)	2 (10%)	4 (20%)	5 (25%)
Soft stool	7 (35%)	9 (45%)	10 (50%)	11 (55%)

QT group, bismuth quadruple therapy for 2 weeks; QF group, bismuth quadruple therapy for 2 weeks combined with fucoidan for 6 weeks; QS group, bismuth quadruple therapy combined with synbiotics for 6 weeks; QFS group, bismuth quadruple therapy combined with both fucoidan and synbiotics for 6 weeks.

### 3.2 Diversity of the gut microbiota before and after the interventions

**Figure 1:**
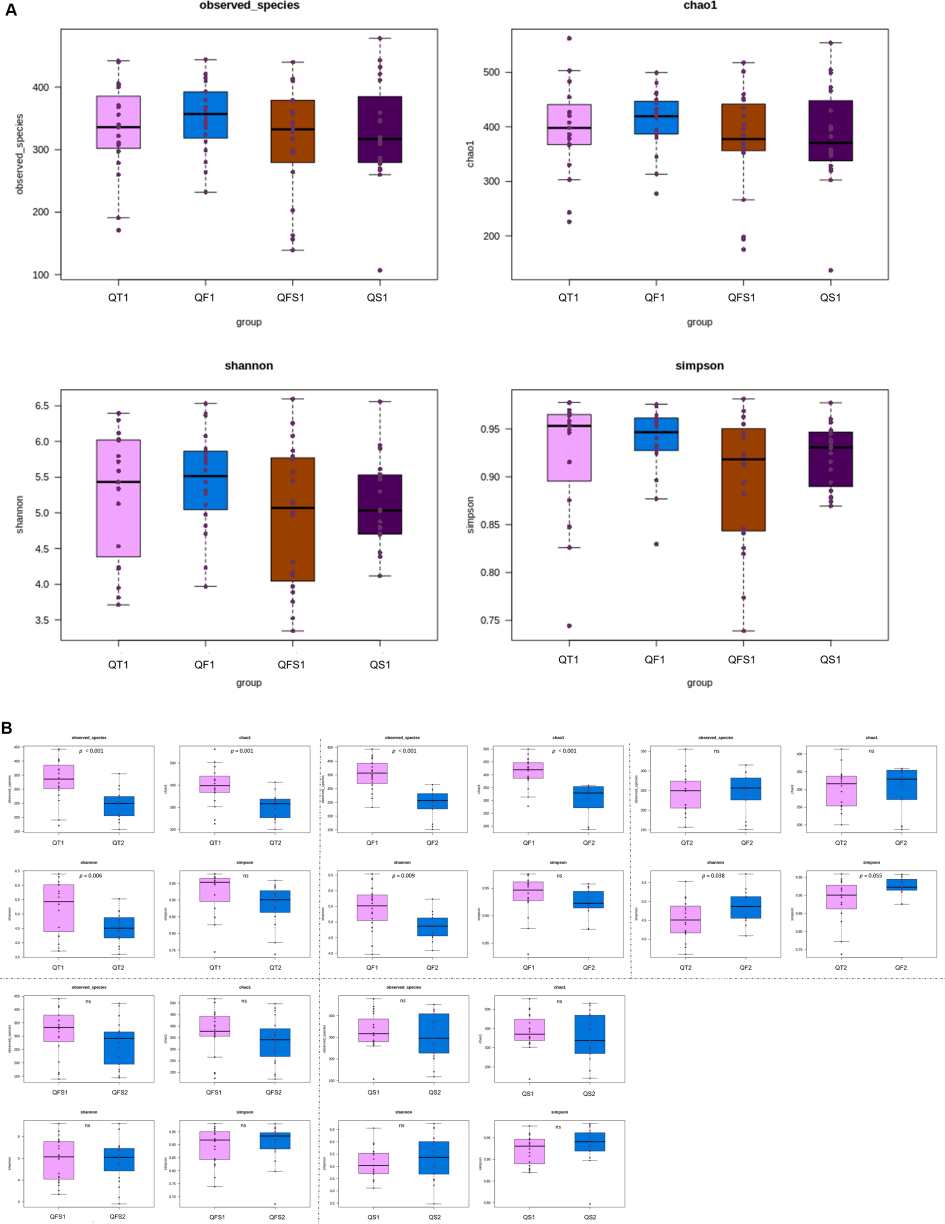
Alpha diversity was depicted with observed species, Chao1, Shannon, and Simpson indices. QT group, bismuth quadruple therapy for 2 weeks; QF group, bismuth quadruple therapy for 2 weeks combined with fucoidan for 6 weeks; QS group, bismuth quadruple therapy combined with synbiotics for 6 weeks; QFS group, bismuth quadruple therapy combined with both fucoidan and synbiotics for 6 weeks. QT1, QF1, QS1, and QFS1 represent the results at baseline; QT2, QF2, QS2, and QFS2 represent the results at the end of week 6. **(A)** No significant differences in the α-diversity were observed among the four groups at baseline. **(B)** The α-diversity significantly decreased in the QT group; Observed species, Chao 1 and Shannon index also significantly decreased in the QF group. No significant differences in the α-diversity were observed in the QS and QFS groups. Compared with the QT group, the Simpson index at week 6 was significantly higher and the Shannon index tended to be higher in the QF group.

All of the patients in the four groups provided fecal samples before the interventions. Four patients in the QT group, 3 patients in the QF group, 3 patients in the QS group, and 1 patient in the QFS group did not provide fecal samples at week 6. There were no significant differences in α-diversity among the four groups at week 0 (Figure 1A). In the QT group, observed species (*p* < 0.001), Chao 1 index (*p* = 0.001), and Shannon index (*p* = 0.006) were all significantly lower at week 6 compared with those at week 0, indicating that the decrease of α-diversity did not restore at 4 weeks after the quadruple therapy. Similarly, significant decreases in observed species (*p* < 0.001), Chao 1 index (*p* < 0.001), and Shannon index (*p* = 0.009) were observed in the QF group. However, these three indices and the Simpson index showed no significant differences between week 0 and week 6 in both the QS and QFS groups. Compared to the QT group, the Simpson index at week 6 was significantly higher (*p* = 0.038) and the Shannon index tended to be higher (*p* = 0.055) in the QF group (Figure 1B).

**Figure 2:**
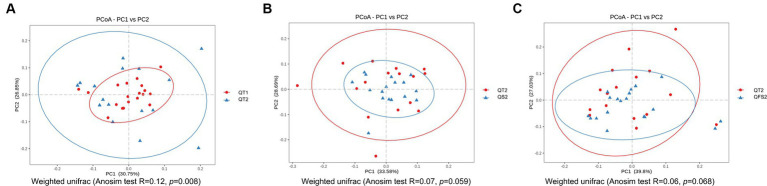
In β-diversity analysis, **(A)** showed that the global microbiota structure in QT group between week 0 and week 6 differed significantly. QT1 represented the results at baseline and QT2 represented the results at the end of week 6. **(B)** showed that the global microbiota structure tended to be different between QT2 and QS2,although statistical significance was not reached. **(C)** showed that the global microbiota structure tended to be different between QT2 and QFS2, although statistical significance was not reached. QT2, QS2 and QFS2 all represented the results at the end of week 6.

In β-diversity analysis, weighted UniFrac PCoA and Anosim showed that the global microbiota structure in QT group between week 0 and week 6 differed significantly (Figure 2A). However, no significant differences in β-diversity were observed before and after interventions in the other three groups. By week 6, the β-diversity of both the QS and QFS groups tended to be different from that of the QT group, although statistical significance was not reached (Figures 2B,C).

### 3.3 Differences in the gut microbiota composition before and after the interventions

**Figure 3:**
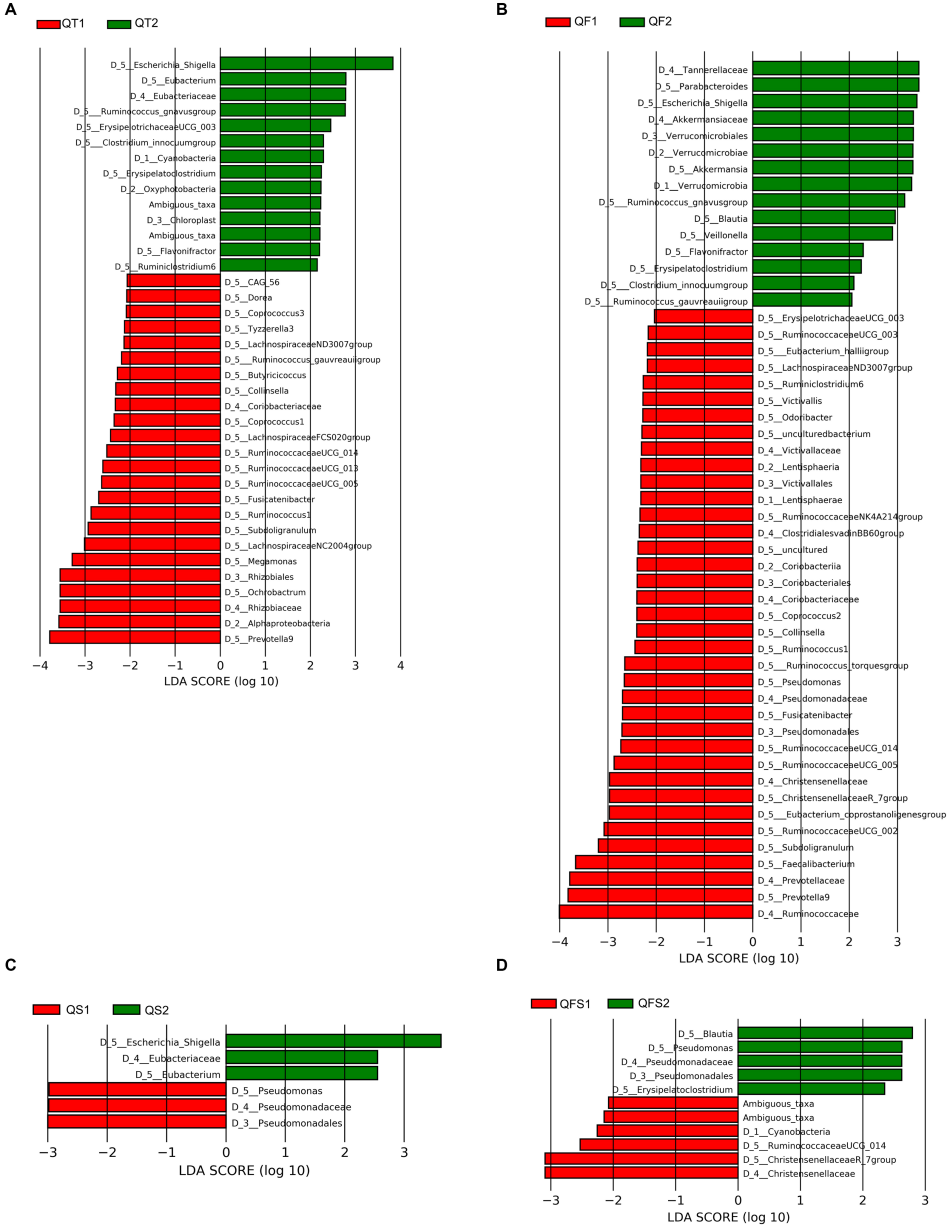
Differences in the gut microbiota composition between week 0 and week 6 in the QT group. **(A–D)** showed differences in the gut microbiota composition between week 0 and week 6 in the QT, QF, QS and QFS group, respectively.

The relative abundance of the four dominant phyla, Firmicutes, Bacteroidetes, Proteobacteria, and Actinobacteria, displayed no significant differences between week 0 and week 6 in the four groups. After 4 weeks of the completion of quadruple therapy, the gut microbiota of the QT group was characterized by the decreased relative abundance of the Alphaproteobacteria class and the Coriobacteriaceae family, as well as the increased relative abundance of the Oxyphotobacteria class, as identified by LEfSe. At the genus level, 28 taxa showed significantly different levels between week 0 and week 6, including 8 increased genera and 20 decreased genera. Most of the decreased genera belong to the Lachnospiraceae family (10 genera, including *Coprococcus 1*, *Coprococcus 2*, *Lachnospiraceae FCS020 group*, *Lachnospiraceae ND3007 group*, *Lachnospiraceae NC2004 group*, *Fusicatenibacter*, *Dorea*, *Tyzzerella 3*, *gauvreauii group*, and *CAG-56*) or Ruminococcaceae (6 genera, including *Butyricicoccus*, *Ruminococcaceae UCG-005*, *Ruminococcaceae UCG-013*, *Ruminococcaceae UCG14*, *Ruminococcus 1*, and *Subdoligranulum*), the others included *Collinsella*, *Megamonas*, *Ochrobactrum*, and *Prevotella 9*. Among the increased genera, *Escherichia-Shigella* contributed most to the differences in the microbiome configuration between week 0 and week 6, with the LDA score (log10) close to 4 (Figure 3A).

In the QF group, the intervention also resulted in significant variations in gut microbiota composition at the genus level. Four genera belonging to Ruminococcaceae (*Subdoligranulum*, *Ruminococcus 1*, *Ruminococcaceae UCG-005*, *Ruminococcaceae UCG-014*) and 3 genera belonging to Lachnospiraceae (*Fusicatenibacter*, *Coprococcus 2*, *Lachnospiraceae ND3007 group*) were significantly decreased in both QT and QF groups at week 6. *Escherichia-Shigella* also increased significantly after the intervention in the QF group. Unlike the QT group, *Parabacteroides*, *Akkermansia*, *Blautia*, and *Erysipelatoclostridium* significantly increased in the QF group at week 6 (Figure 3B).

The results of LEfSe analysis demonstrated that patients in the QS and QFS groups experienced relatively slight microbiota perturbation during the interventions. *Escherichia-Shigella* significantly increased in the QS group but not in the QFS group. The Pseudomonadaceae family and *Pseudomonas* significantly increased in the QFS group while significantly decreased in the QS group. Eubacteriaceae family and *Eubacterium* were significantly more abundant in the QS group after the intervention. Additionally, in the QFS group, *Blautia* and *Erysipelatoclostridium* became significantly more abundant while the Christensenellaceae family became significantly less abundant at week 6 (Figures 3C,D).

### 3.4 Differences in gut microbiota composition among the groups

To further detect the effects of fucoidan and symbiotic supplementation on the variation of microbiota in the context of quadruple therapy, we used LEfSe and Metastats to compare the microbiota taxa between the QT group and the QF, QS, or QFS groups, respectively.

**Figure 4:**
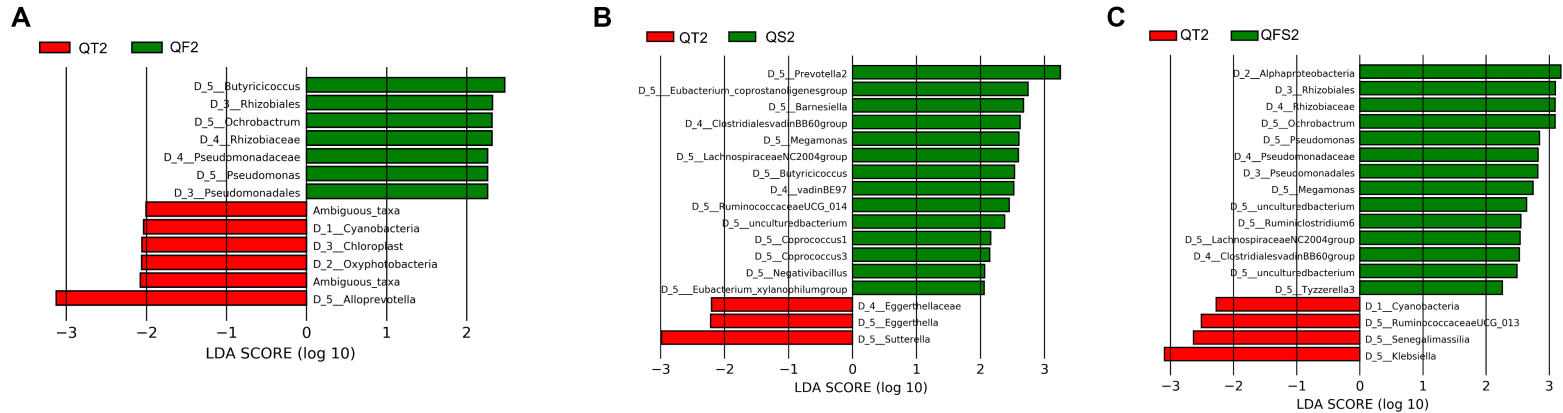
**(A)** showed differences in the gut microbiota composition between QT group and QF group after the intervention. **(B)** showed differences in the gut microbiota composition between QT group and QS group after the intervention. **(C)** showed differences in the gut microbiota composition between QT group and QFS group after the intervention.

Between the QT and QF groups, LEfSe analysis revealed that *Butyricicoccus* in Ruminococcaceae family and *Ochrobactrum* in Rhizobiaceae family were significantly more abundant in the QF group at week 6, while their relative abundances were not significantly different between the two groups at week 0 (Figure 4A). Metastats also confirmed that the level of *Butyricicoccus* was significantly higher in the QF group at week 6.

Between the QT and QS groups, LEfSe showed that 4 genera belonging to Ruminococcaceae (*Butyricicoccus*, *Negativibacillus*, *RuminococcaceaeUCG-014*, *coprostanoligenes group*), 4 genera belonging to Lachnospiraceae (*LachnospiraceaeNC2004group*, *Coprococcus 1*, *Coprococcus 3*, *xylanophilum group*), *Prevotella 2* in Prevotellaceae, *Megamonas* in Veillonellaceae, and *Barnesiella* in Barnesiellaceae had significantly higher levels in QS group at week 6, while the relative abundance of *Negativibacillus*, *Prevotella 2*, *LachnospiraceaeNC2004group*, *xylanophilum* group did not reach 0.01% (Figure 4B). Metastats also supported that *Butyricicoccus*, *RuminococcaceaeUCG-014*, and *Megamonas* were significantly more abundant in the QS group at week 6.

Between the QT and QFS groups, LEfSe found that the relative abundance of *Ochrobactrum* and *Megamonas* significantly increased in the QFS group. Among the genera with a relative abundance of over 0.01%, *Tyzzerella 3* and *Ruminoclostridium 6* were significantly more abundant while *Klebsiella* was significantly less abundant in the QFS group (Figure 4C). In parallel, Metastats confirmed the higher abundance of *Ochrobactrum* in the QFS group and detected that *Butyricicoccus* was significantly more abundant in the QFS group. The comparison of *Butyricicoccus* in the two groups at week 6 showed a trend of a higher relative abundance of *Butyricicoccus* in the QFS group, but statistical significance was not reached. Table 3 shows the relative abundance of the genera mentioned above before and after different interventions, displaying the variations in gut microbiota across different groups.

**Table 3:** Comparison of the relative abundance of genera between the quadruple therapy group and the groups with fucoidan and/or symbiotic.

Bacterial genera	Before intervention (week 0)			After intervention (week 6)		
	QT group	QF group	*p*	QT group	QF group	*p*
*Butyricicoccus*	0.223% ± 0.197%	0.247% ± 0.166%	NS	0.087% ± 0.119%	0.384% ± 0.387%	0.022
*Ochrobactrum*	3.639% ± 5.192%	1.004% ± 3.208%	NS	0.029% ± 0.070%	0.221% ± 0.450%	0.028
	QT group	QS group	*p*	QT group	QS group	*p*
*Butyricicoccus*	0.223 ± 0.197%	0.311% ± 0.287%	NS	0.087 ± 0.119%	0.384 ± 0.401%	0.011
*RuminococcaceaeUCG-014*	2.981% ± 5.928%	2.899% ± 6.572%	NS	0.000% ± 0.000%	2.183% ± 4.576%	0.034
*Megamonas*	2.246% ± 4.913%	0.716% ± 2.212%	NS	0.002% ± 0.003%	0.351% ± 1.191%	0.034
*Coprococcus 1*	0.029 ± 0.030%	0.039% ± 0.049%	NS	0.009% ± 0.020%	0.069% ± 0.108%	0.034
*Coprococcus 3*	0.059% ± 0.102%	0.036% ± 0.029%	NS	0.012% ± 0.038%	0.060% ± 0.069%	0.028
*Coprostanoligenes group*	0.527% ± 1.392%	0.421% ± 0.696%	NS	0.378% ± 1.186%	0.811% ± 1.463%	0.023
*Barnesiella*	0.518% ± 0.780%	0.512% ± 0.972%	NS	0.257% ± 0.670%	0.696% ± 1.314%	0.041
	QT group	QFS group	*p*	QT group	QFS group	*p*
*Butyricicoccus*	0.223% ± 0.197%	0.279% ± 0.294%	NS	0.087% ± 0.119%	0.356% ± 0.421%	0.058
*Ochrobactrum*	3.639 ± 5.192%	0.936% ± 2.527%	NS	0.029% ± 0.070%	1.158% ± 2.862%	0.002
*Megamonas*	2.246% ± 4.913%	0.683% ± 2.673%	NS	0.002% ± 0.003%	0.454% ± 1.047%	0.043
*Tyzzerella 3*	0.061% ± 0.106%	0.072% ± 0.190%	NS	0.036% ± 0.144%	0.129% ± 0.503%	0.048
*Ruminoclostridium 6*	0.061 ± 0.194%	0.332% ± 0.649%	NS	0.073% ± 0.293%	0.318% ± 1.260%	0.020
*Klebsiella*	2.407% ± 2.805%	0.960% ± 2.056%	NS	1.204% ± 1.967%	0.273% ± 0.945%	0.009

The data are presented as the mean ± SD; QT group, bismuth quadruple therapy for 2 weeks; QF group, bismuth quadruple therapy for 2 weeks combined with fucoidan for 6 weeks; QS group, bismuth quadruple therapy combined with synbiotics for 6 weeks; QFS group, bismuth quadruple therapy combined with both fucoidan and synbiotics for 6 weeks; NS, no significance.

## 4 Discussion

To our knowledge, this study is the first to report the effects of fucoidan combined with synbioticss on the gut microbiota of *H. pylori*-infected patients treated with bismuth quadruple therapy. We comprehensively compared the gut microbiota before and after 4 regimens of therapy in patients with *H. pylori* infection. As anticipated, quadruple therapy led to significant variations in the microbiome configuration. Although the α-diversity was also decreased in the QF group, a significantly higher level of Simpson index was observed in the QF group compared with the QT group, and β-diversity did not change significantly in the QF group. In contrast, analyses of α-diversity and β-diversity demonstrated that the microbiota composition after the 6 weeks of interventions had no significant alteration compared with baseline in the QS group and the QFS group, with only a few bacterial taxa significantly changed in relative abundance. The supplementation of fucoidan or synbioticss might promote the growth of *Butyricicoccus*, evidenced by the significantly higher level of *Butyricicoccus* in all of the QF, QS, and QFS groups after the interventions. Moreover, the combination of fucoidan and synbioticss might inhibit the growth of *Escherichia-Shigella*, supported by enriched *Escherichia-Shigella* in the QT, QF, and QS groups except for QFS group.

The perturbation of microbiota caused by eradication therapy has attracted attention in *H. pylori* infection-related research. It has been demonstrated that the significant reduction of α-diversity of gut microbiota after bismuth quadruple therapy may not be restored even a year after the therapy. This phenomenon was also observed in β-diversity (20). Our results also supported striking variations in α-diversity and β-diversity following quadruple therapy. Chen L et al. found that a 14-day bismuth quadruple therapy induced a significant decrease in the relative abundance of Lachnospiraceae and Ruminococcaceae, in which the stool samples were collected at the end of the therapy (21). Different from Chen’s study, the fecal samples were collected 4 weeks after completion of the quadruple therapy in our study, yet we still observed that most of the decreased genera belonged to Lachnospiraceae or Ruminococcaceae, and the relative abundance of the Coriobacteriaceae family was also significantly decreased. Evidence is accumulating that Lachnospiraceae and Ruminococcaceae contribute to the production of SCFAs from dietary fibers in the gut (22, 23). SCFAs can modulate both innate and adaptive immunity by inhibiting cytokines and regulating migration and differentiation of immune cells (24). Moreover, SCFAs can enhance the expression of tight junction proteins in the intestinal epithelium and help maintain the integrity of the colonic mucus layer, playing an important part in gut barrier function (25, 26). Among the three major SCFAs, namely acetate, propionate, and butyrate, butyrate serves as the main energy source for colonocytes and can inhibit the growth of pathogens by modulating luminal pH (27). It has been reported that genera such as *Butyricicoccus* (28), *Coprococcus* (29), and *Subdoligranulum* (30) can all exert butyrate-producing function. Besides, *Dorea*, *Collinsella*, and *Fusicatenibacter* were found to increase in patients with Crohn’s disease or functional constipation after fecal microbiota transplantation, in parallel with an increase of fecal butyrate (31, 32), suggesting the correlation between these genera and butyrate production. Notably, all of the 6 genera mentioned above significantly decreased in the QT group, indicating the inhibition of growth of these beneficial genera and potential disturbance of SCFAs production caused by bismuth quadruple therapy. In agreement with previous studies (33, 34), we also observed that the opportunistic pathogen in Enterobacteriaceae, *Escherichia-Shigella*, significantly increased after the bismuth quadruple therapy (35). *Escherichia-Shigella* may exert a pro-inflammatory effect on the host by increasing serum Lipopolysaccharide (36), potentially promoting low-grade systematic inflammation.

In the present study, fucoidan exhibited the potential in preserving the α-diversity of the gut microbiota. As a soluble dietary fiber, fucoidan from *Laminaria japonica* can be fermented by the gut microbiota of human and increase the level of SCFAs and several probiotics, such as *Bifidobacterium*, *Lactobacillus*, and *Enterobacter* (37), indicating its prebiotic properties. Animal experiments have shown that fucoidan can significantly increase the α-diversity of microbiota, ameliorate dyslipidemia, protect gut barrier function, and decrease systematic inflammation (38, 39). Our results indicated that the reduction in intestinal microbiota diversity caused by quadruple therapy can be reversed partially by fucoidan. Patients in the QF group also experienced perturbations in gut microbiota, primarily at the genus level. However, different from the QT group, several beneficial genera became significantly more abundant in the QF group after the intervention, including *Butyricicoccus*, *Akkermansia*, *Blautia*, and *Parabacteroides.* Shi et al. reported that fucoidan supplementation could increase the levels of *Butyricicoccus* as well as fecal SCFAs and SCFA receptors in the small intestine (40). Additionally, there is also evidence showing that fucoidan can increase the levels of *Blautia*, *Akkermansia*, and *Parabacteroides* (41, 42). *Blautia* and *Parabacteroides* can exert the function of SCFAs-production (43, 44), while *Akkermansia* has been reported to protect the gut barrier function by modulating the number of goblet cells (45). Moreover, *Erysipelatoclostridium* significantly increased in the QF group compared with baseline level, and *Ochrobactrum* significantly increased in the QF group at week 6 compared to the QT group. Interestingly, swimming can induce the increase of SCFAs and *Erysipelatoclostridium* in feces of colitis-associated cancer mice (46), and fecal *Ochrobactrum* significantly increased after the butyrate supplement in mice (47), indicating that a gut environment with more SCFAs might promote the growth of *Erysipelatoclostridium* and *Ochrobactrum*. Collectively, the detrimental effects of bismuth quadruple therapy on gut microbiota, especially on some SCFAs-producing taxa, may be partly mitigated by fucoidan.

Synbioticss, composed of both probiotics and prebiotics, may have superior effects compared to probiotics or prebiotics alone, owing to enhanced probiotic survival in the gastrointestinal tract and stimulation of native beneficial bacterial proliferation by prebiotics (48). Strains of *Lactobacillus* and *Bifidobacterium* are mainstream probiotics and have been proven safe and effective, capable of not only producing lactic acid and acetic acid but also promoting the growth of some butyrate-producing bacteria (49). Our findings indicate that synbioticss could protect patients from the harmful effects of bismuth quadruple therapy on the richness and stability of gut microbiota. Moreover, compared to the QT group, the levels of the beneficial genera mentioned above, such as *Butyricicoccus* and *Coprococcus*, were significantly higher after the daily use of the synbiotics for 6 weeks. Additionally, *Megamonas* and *Barnesiella* also significantly increased in the synbiotics group. *Megamonas* is reported as SCFAs-producer (50, 51), and there is evidence supporting that the supplement of dietary fiber may facilitate the growth of *Megamonas* (52). *Barnesiella* may help to treat infections caused by antibiotic-resistant bacteria (53) and ameliorate oxidative stress levels (54). Moreover, when comparing to the level before intervention, the relative abundance of *Eubacterium* significantly increased in the synbiotics group, which is a major butyrate producer and participates in the modulation of gut inflammation and maintenance of gut barrier integrity (55). It is worth noting that the addition of fucoidan or synbiotics to bismuth quadruple therapy did not prevent the increase of *Escherichia-Shigella*, whereas the combined use of these two supplementations showed a promising effect on inhibiting the growth of *Escherichia-Shigella*. Another interesting finding is that the relative abundance of *Klebsiella* significantly decreased in the group using both fucoidan and synbioticss. Same as *Escherichia-Shigella*, *Klebsiella* spp. are also categorized within Enterobacteriaceae family and are recognized as opportunistic pathogens, with *Klebsiella pneumoniae* being the most notable one (56). Therefore, in terms of the inhibition of potential pathogens, the combined administration of synbiotics and fucoidan might offer a significant advantage over synbiotics supplementation alone.

In this study, supplementation of fucoidan, synbioticss, or their combination showed no improvement in the eradication rate of bismuth quadruple therapy. Fucoidan has been reported to inhibit the growth of *H. pylori* and disrupt the adherence of *H. pylori* to gastric epithelial cells (57). Besides, fucoidan has been demonstrated to dampen inflammation in the stomach of *H. pylori*-infected mice (58). However, the application of fucoidan in patients with *H. pylori* infection is limited, and there has been no convincing evidence supporting the efficacy of fucoidan in increasing the eradication rate of *H. pylori* infection. In terms of synbioticss, a meta-analysis showed that in per-protocol analysis, the eradication rate had no significant improvement when adding synbioticss to *H. pylori* eradication therapy, despite that pooled effect size from intention-to-treat analysis supported the role of synbioticss in the eradication of *H. pylori* (59). It should be noted that the eradication rate in the group only used RMAB therapy in our study reached 95.0%, consistent with a previous study showing that the eradication rate of RMAB therapy was 92.6% in per-protocol analysis (60). Therefore, the satisfactory eradication rate of RMAB therapy may leave little space for us to observe the effects of the supplementations on *H. pylori* eradication.

There were certain limitations in this study. Firstly, the variations in gut microbiota induced by fucoidan may vary with the dosage of fucoidan (16), thus it is necessary to compare the effects of different doses of fucoidan in *H. pylori-*infected patients treated with bismuth quadruple therapy in a future study, and more beneficial functions of fucoidan may be found besides those on the gut microbiota. Secondly, since the most significant perturbation of gut microbiome may occur at the end of bismuth quadruple therapy (i.e., week 2), collecting stool samples at this time point would provide further insight into the protective role of fucoidan and synbioticss in maintaining intestinal microecology homeostasis. Lastly, multi-center studies with larger sample sizes are required to validate the findings of this study.

## 5 Conclusion

This study showed that the gut microbiota of patients with *H. pylori* infection had remarkable changes after bismuth quadruple therapy, especially the increase in the relative abundance of *Escherichia-Shigella* and decrease in certain genera of Lachnospiraceae and Ruminococcaceae families. Fucoidan supplementation might ameliorate the adverse effects of quadruple therapy on the diversity of intestinal microbiota. The synbiotics containing *Bifidobacterium*. spp., *Lactobacillus*. spp. and dietary fibers could help to maintain the stability of microbiota composition. Fucoidan or synbiotics supplementation might help to increase the relative abundance of several beneficial taxa. Notably, combined supplementation of fucoidan and the synbiotics packet showed the potency to inhibit the growth of potential pathogens including *Escherichia-Shigella* and *Klebsiella*, which might be a promising adjuvant regimen to alleviate gut dysbiosis during *H. pylori* eradication therapy.

## Data availability statement

The raw data supporting the conclusions of this article will be made available by the authors, without undue reservation.

## Ethics statement

The studies involving humans were approved by the Ethics Committee of China-Japan Friendship Hospital. The studies were conducted in accordance with the local legislation and institutional requirements. The participants provided their written informed consent to participate in this study.

## Author contributions

HW: Formal analysis, Investigation, Methodology, Writing – review & editing. WW: Formal analysis, Investigation, Writing – original draft. FL: Data curation, Validation, Writing – original draft. MW: Investigation, Writing – original draft. YZ: Conceptualization, Supervision, Validation, Writing – review & editing. SD: Conceptualization, Funding acquisition, Project administration, Resources, Writing – review & editing.
